# Trick or Treat: The Role of Vaccines in Integrated Schistosomiasis Control

**DOI:** 10.1371/journal.pntd.0000244

**Published:** 2008-06-25

**Authors:** Robert Bergquist, Jürg Utzinger, Donald P. McManus

**Affiliations:** 1 Ingerod, Brastad, Sweden; 2 Department of Public Health and Epidemiology, Swiss Tropical Institute, Basel, Switzerland; 3 Molecular Parasitology Laboratory, The Queensland Institute of Medical Research, Brisbane, Australia; Queensland Institute of Medical Research, Australia

## Neglected and Underappreciated—The Case of Schistosomiasis

Recent systematic reviews [1],[2] indicate that the geographic extent and burden of schistosomiasis exceeds official estimates. The risk for infection is particularly pronounced in sub-Saharan Africa, but also exists in many parts of South America, the Middle East, and Southeast Asia. Collectively, close to 800 million individuals are at risk of schistosomiasis, and over 200 million people are infected [2]. The disease can be controlled with vigorous political and financial commitment, but local elimination has proved difficult in reality. The cost of treatment with the only available drug—praziquantel—has become affordable, and hence preventive chemotherapy is being advocated on a large scale [3]. However, a strongly biased approach, devoid of any emphasis on prevention, access to clean water and improved sanitation, and snail control, demands indefinite drug distribution [4],[5]. Although praziquantel-based morbidity control has proved successful, it has an intrinsic weakness, which is intimately related to its inadequate impact on transmission even when chemotherapy is provided according to schedules carefully adjusted to the local setting [6].

Genuine change of the disease spectrum in endemic settings demands lasting results which can only be obtained by long-term protection involving vaccination. An entirely vaccine-based approach to schistosomiasis control is unrealistic, but we advocate that acceptable protection could be achieved by chemotherapy followed by vaccination aimed at reducing, or markedly delaying, the development of pathology [7]. Thus, the issue is not vaccines *versus* chemotherapy, but how to graft a vaccine approach onto current schistosomiasis control programs. This, we believe, would fit well with the scope of the “Schistosomiasis Research Agenda”, particularly in the areas of “Basic Science”, “Tools and Interventions”, and “Disease Burden” advanced by Colley and Secor in their recent Policy Platform article in *PLoS Neglected Tropical Diseases* [8] and the linked Expert Commentary [9].

## The True Impact of Schistosomiasis

Currently, vaccines do not figure prominently in the context of schistosomiasis control. In fact, neither vaccines nor new drug development are pursued with high priority by funding bodies, industry, or academia. There are two important reasons for this. First, praziquantel is safe, efficacious, and inexpensive (recently even donated free of charge in some control programs) and, as yet, there are no clear indications of drug resistance. Second, a low rank is awarded to schistosomiasis by the disability-adjusted life year (DALY) metric, an approach guiding the policies of most organizations active in the public health area, including the World Health Organization (WHO) [10],[11].

**Figure 1:**
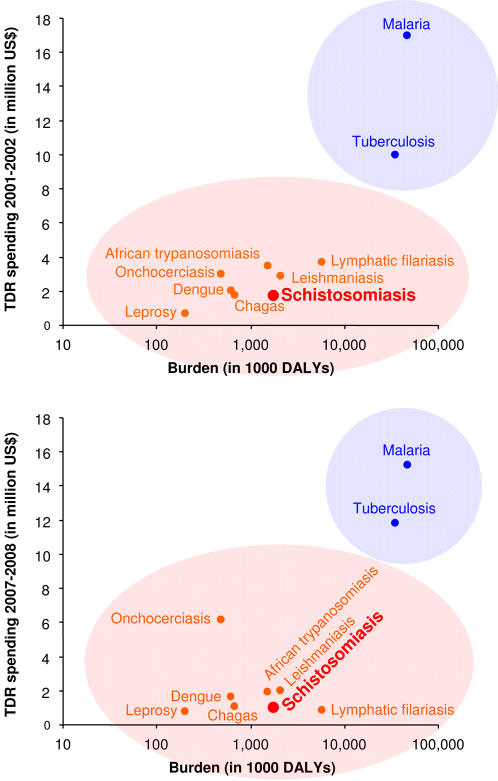
Yardstick for Financial Resource Allocation Based on the Global Burden Awarded to Each Disease in TDR's Portfolio in the 2001–2002 Biennium (Top) and the 2007–2008 Biennium (Bottom). The figures show that the diseases fall into two relatively distinct groups, (i) well-funded (i.e., malaria and tuberculosis), and (ii) less well-funded, including leprosy, which is now targeted for elimination as a threat in public health. Note the particularly low amounts currently allocated to schistosomiasis and lymphatic filariasis in relation to the estimated global burden of these diseases.

Figure 1 illustrates the impact according to the DALY metric of the ten diseases supported by the UNICEF/UNDP/World Bank/WHO Special Programme for Research and Training in Tropical Diseases (TDR) in relation to research spending for the biennium 2001–2002 [7] and 2007–2008 [12]. The difference between the two graphs in Figure 1 seems to indicate a move away from a rigorous adherence to the DALY metric as the key to funding that was evident at the beginning of the new millennium. Indeed, there are several “outliers” in the latest TDR budget, which could be due to a desire to make a difference where needed (e.g., onchocerciasis), while control successes in other areas may have given the impression that research is less needed there (e.g., schistosomiasis and lymphatic filariasis). In our opinion, and that of others [1], the low ranking of schistosomiasis does not appear to be justified. First, research aimed at the long-term control of schistosomiasis remains important despite current successes. Second, if all health problems due to this disease were considered, it should actually be in the top category together with malaria and tuberculosis [1],[13]. Currently, the DALY measure for schistosomiasis only includes overt signs of morbidity, while more common sequelae such as anemia, growth retardation, impaired cognitive development, and exercise intolerance are not [1],[14]. In addition, future re-calculations of the global burden of schistosomiasis and other neglected tropical diseases (NTDs) should also consider the likely influence of co-infection and co-morbidity [14],[15], and their relation to health promotion strategies in endemic areas. Most important from the control point of view, however, is the observation that the recurrent aggressive inflammation following interrupted chemotherapy in 80% of children living in high-transmission areas [16] requires frequent praziquantel treatment to avoid making the situation worse than it might have been without any intervention at all. This inflammatory response, due to interference with the immunological modulation that normally takes place during the course of natural infection, severely exacerbates pathology in epidemiological settings where treatment schedules cannot be sustained.

## Contemporary Control Emphasis

Current funding for schistosomiasis is far from negligible, but most of the money is allocated for a single approach: the procurement, distribution, and deployment of praziquantel. Over the past five years, the Schistosomiasis Control Initiative (SCI; http://www.schisto.org/), which is funded by the Bill & Melinda Gates Foundation, has spent close to US$50 million for mass drug administration targeting schistosomiasis and soil-transmitted helminthiases in six African countries. Although SCI is the architect of one of the great control successes of modern times, and has been awarded the Queen's 2007 Anniversary Prize, the solution is incomplete because transmission is only marginally affected and the long-term outlook limited by re-infection [6],[15].

SCI will be continued, and its scope expanded and increasingly integrated with control efforts targeting other NTDs, supported by additional funding from multiple sources. Collaborative work in this field includes a US$8.9 million grant from Geneva Global awarded to the Global Network for Neglected Tropical Disease Control (GNNTDC; http://gnntdc.sabin.org/) and a US$100 million grant from the United States Agency for International Development (USAID) awarded to RTI International (http://www.rti.org/) with the goal of treating 40 million individuals over a 5-year period [17]. Most significantly, a US$350 million new global initiative to combat NTDs was announced by US President George W. Bush earlier this year [18].

## Is Schistosomiasis Vaccine Development Justified?

It could be argued that the success of SCI constitutes the justification needed for vaccine development, since an effective vaccine would add the necessary long-term perspective presently lacking in schistosomiasis control strategies. Moreover, the repositioning of vaccines as the solution to a chemotherapy problem would be a novel, revitalizing concept in a field where control activities have remained exclusively focused on morbidity reduction for too long. The revised strategy would combine immediate opportunity with the need for longer term support of ongoing discovery and skills development.

Resistance against praziquantel in the future cannot be ruled out even if, after more than 20 years of large-scale application in some places, there is still no clear-cut evidence pointing in that direction. However, if (when) resistance does occur, a replacement drug must be available for immediate implementation. Otherwise, not only would the current strategy collapse, but the integrated drug–vaccine approach that we are advocating here would also be seriously impaired. It is therefore worrisome, from both of these points of view, that praziquantel is the only drug available at this juncture.

In contrast to the time before the advent of recombinant techniques, we now have the tools to produce particular biological materials in large amounts at a reasonable cost. In addition, the complementary approach, bypassing antigen preparation and relying on injection of DNA itself, has gained wide acceptance. Thanks to this technical progress, industrial vaccine production must now be deemed realistic, while the arguments supporting the possibility of succeeding within the field of schistosomiasis remain strong for the following reasons:

- humans living in endemic areas develop some degree of protection naturally with some becoming immune [19],[20];
- irradiated cercariae confer >80% protection in experimental animal models [21];
- schistosomiasis vaccine candidates already exist, which produce ≥40% protection in animal models [22]; and
- highly efficacious recombinant veterinary vaccines against taeniid cestodes have been developed [23].

The linking of vaccination with chemotherapy would reduce overall morbidity and limit the impact of re-infection. Therefore, although induction of consistent, high-level protection has not been recorded for any of the available candidate vaccine antigens, the commonly reported protection levels of ∼40%–50% in experimental animals [24] should, in our opinion, be sufficient for a combined drug–vaccine approach to improve significantly on the current strategy, which is based on chemotherapy alone.

Vaccine development so far has been focused on *Schistosoma mansoni*, resulting in progress towards Phase I safety trials of one vaccine candidate, the 14-kDa fatty acid-binding protein (Sm14) [25]. The other vaccine candidate to have entered clinical trials, a 28-kDa glutathione-*S*-transferase (Sh28GST, also known as BILHVAX), is derived from *S. haematobium* [26], a schistosome species otherwise neglected with regard to vaccine development, but which clearly is important for future study, particularly as most human cases of schistosomiasis are due to *S. haematobium* [27]. For *S. japonicum*, a number of promising vaccine candidates already exist [22]. The fact that *S. japonicum* infects a wide spectrum of final hosts is an added challenge for control programs [28], but is positive from the point of view of vaccine development, since it permits a step-wise tactic that would start with a “transmission-blocking” veterinary product [29] before moving on to the human vaccine. The possibility that this strategy could pay off already before a human vaccine is realized is supported by Chinese studies showing that the animal–snail–human transmission cycle is more prominent than the human–snail–human one in sustaining the infection [30],[31].

## New Approaches in Antigen Discovery

The current *Schistosoma* vaccine candidates may prove not to be the most effective, and it is, therefore, important to continue to identify new target antigens. The generation of a large schistosome transcriptome database and postgenomic technologies, including DNA microarray profiling, proteomics, glycomics, and immunomics, offer the necessary ancillary information [22], [32]–[34]. These new approaches in antigen discovery have the potential to identify a new generation of vaccine target molecules that may induce greater potency than the current candidate schistosome antigens [22]. Molecules containing signal peptides and signal anchors as predictors of excretory-secretory products, including enzymes and components exposed on the schistosome tegument (including receptors) that interact directly with the host immune system, are highly relevant targets for study [22],[34].

## Human Immune Responses to Vaccine Candidate Antigens

The analysis of human antibody and cytokine responses to candidate vaccine antigens is a creditable way for establishing bona fide vaccine candidates. Such a study, focusing on the ten most promising schistosome vaccine antigens, was carried out over several years by the Egyptian Reference Diagnostic Center in Cairo in the 1990s [35]. At various time points, immune responses against a panel of antigens were determined in cohorts of humans living in areas where they were regularly exposed to infection and these results compared with parasitological diagnosis. Cellular and humoral immune responses were significantly associated with either apparent resistance or with apparent susceptibility to re-infection following chemotherapy. However, only a minority of these responses produced consistent associations and the results were seldom clear-cut. A similar investigation, carried out in the Philippines [36], confirmed the Egyptian findings, but a straightforward comparison was not possible because some of the antigens tested were different in the two studies. The discovery of the surface-located tetraspanins Sm-TSP-1 and TSP-2 as candidate vaccine antigens resulted from a combination of protective efficacy data obtained in the *S. mansoni* murine challenge model with their recognition by IgG1 and IgG3 antibodies from humans exposed but resistant to schistosomiasis [37]. These human studies are instructive for not only identifying the few antigens directly and exclusively associated with resistance, but also for indicating which of these components can be formulated with adjuvants to generate protective responses in animal models.

## Concluding Remarks

Protection against schistosomiasis should not only reduce infection and protect from re-infection, but also accelerate immune responses in infected humans directed against granuloma-related pathology and/or worm fecundity [22]. However, the funding needed for a revised control strategy will clearly not be forthcoming without re-emphasizing the clinical dimension. Thus, rigorous re-calculation of the true burden and societal impact of schistosomiasis, in the light of the new data on mortality and morbidity (including “subtle pathologies”), is urgently needed [1],[13],[14].

There is no denying that schistosomiasis vaccine development has followed a long and tortuous road [22],[24],[38]. However, the two vaccine candidates that have progressed the furthest toward clinical development—Sh28GST (at the Phase II level) [26] and Sm14 (about to initiate Phase I) [25]—demonstrate that this road is negotiable.
